# Editorial: Environmental risk factors and psychosomatic disorders

**DOI:** 10.3389/fpsyt.2025.1751096

**Published:** 2026-01-05

**Authors:** Peng Yu, Bin Yu, Wanjie Tang, Shujuan Yang

**Affiliations:** 1 West China School of Public Health and West China Fourth Hospital, Sichuan University, Chengdu, China; 2 Institute for Disaster Management and Reconstruction, Sichuan University-The Hong Kong, Chengdu, China; 3 Health Promotion and Food Nutrition & Safety Key Laboratory of Sichuan Province, Chengdu, China; 4 International Institute of Spatial Health Epidemiology (ISLE), Wuhan University, Wuhan, China

**Keywords:** environmental pollution, environmental risk (ER), environmental risk factors, psychosomatic disorders, social environmental factors

Psychosomatic disorders are traditionally defined as a distinct group of conditions primarily driven by stress and psychological distress. These disorders are influenced by environmental exposures (environmental pollution), and social environmental factors (social structures, cultural norms, gender roles, and behavioral settings). This Research Topic aimed to synthesize current evidence on the associations between environmental risk factors and psychosomatic disorders, and to explore pathways for effective prevention and intervention.

## The evolving paradigm of environment in psychosomatic research

The conceptualization of “environment” in psychosomatic research has undergone evolution, moving beyond primarily focusing on air pollutants and toxic substances (1–4) to encompass a far more complex landscape of risk, such as social structures (5–7), family stability (8, 9), cultural norms (10, 11), lifestyle choices (12), and community safety (13). This expanded conception is essential because these diverse environmental factors influence the susceptibility to psychosomatic disorders through stress-physiology-behavior mechanism (14, 15) involving stress appraisal (16), autonomic and neuroendocrine dysregulation, immune activation, and behavioral adaptation (14–18). For example, the link between air pollution and depression may be attributed to the imbalance of neurotransmitters through the central nervous system stress mechanism (19). In addition, built environment, neighborhood socioeconomic status, walkability, and greenness, are significantly associated with symptoms of generalized anxiety disorder (20). These findings highlight the important role of environmental factors in the risk of psychosomatic disorders.

## Evidence from observational studies

From the perspective of the social-ecological model (21, 22), various dimensions of the social environment, encompassing individual, familial, and social factors, serve as chronic stressors, inducing sustained psychosomatic disorders (23). Thus, considering multifaceted social environmental factors into the risk of psychosomatic disorders is fundamental to advancing etiological understanding and formulating comprehensive public health strategies. Evidence from the presented studies, from individual to societal factors, establishes a clear link between these environments and psychosomatic health.

First, socio-cultural structures and family environment are known to influence the development of psychosomatic disorders. A cross-sectional study by Gao et al., indicated that women from the matrilineal Mosuo society showed a milder menopausal symptom, as measured by the Kupperman Menopause Index, and a lower score for concerns and fears about illness on the Self-Rating Scale of Illness Conception and Health Seeking Behavior, compared to women from patrilineal Han and Yi societies. This suggested that that cultural systems affording women higher social status and robust support networks may buffer against the psychosomatic manifestations associated with physiological transitions. Meanwhile, a large-scale, multicenter cross-sectional study by Wei et al., using Rome IV criteria, reported that separation from father only was an independent risk factor for irritable bowel syndrome in rural Chinese left-behind children, with a stronger association than separation from mother. This finding suggested that rural-to-urban migration was a critical environmental risk for psychosomatic disorders, potentially mediated through gut-brain interactions in a vulnerable pediatric population. these findings illustrate socio-cultural and familial factors could elicit psychological distress, and ultimately led to specific somatic symptoms (24–26).

Second, community violence operates as a pervasive psychosocial stressor with broad impacts. One study conducted by Iktilat et al., showed a direct positive association between exposure to violence, measured by the Screen for Adolescent Violence Exposure questionnaire, and psychological distress, assessed by the Kessler 6 scale, in middle-aged Muslims in Israel. This association was significant for both men and women after adjusting for age, education level, socioeconomic status, suggesting that the psychological impact was pervasive across genders in high-violence environments. This finding indicates that violent environments, as intense psychological stressors, exert widespread detrimental effects on individual psychosomatic health (27, 28).

Third, modern lifestyles, such as night-shift work and excessive smartphone use, has emerged as an important risk environment for psychosomatic health. Guo et al. showed a potential causal relationship between various lifestyles and major depressive disorder using Mendelian randomization approach, identifying mood swings, weekly usage of mobile phone and sleeplessness/insomnia as significant risk factors. This study suggests that modern lifestyle factors (e.g., insomnia, excessive phone use, and diet) represent inherent modern behaviors that act as both a source and manifestation of chronic stress, which in turn causes psychosomatic disorders like depression via mechanisms including circadian rhythm disruption (29, 30).

## Outlook for future research

The evidence presented in this Research Topic marks an advancement of psychosomatic disorders in our understanding. From cross-sectional study to longitudinal study, this topic has established the associations between environmental factors and psychosomatic disorders. The findings reveal that the risk factors of psychosomatic disorders are multifaceted, including cultural context, social adversity (separation and violence), and lifestyle behaviors.

These studies advocate for adopting a broader and more inclusive definition of environmental risk in the field of psychosomatic medicine. They demonstrated that the risk factors for psychosomatic disorders is across multiple levels, including sociocultural, community, and individual domains (31). The present findings further validate the pivotal role of the sociocultural environment and underscore the necessity of culturally adapted interventions.

Future research would leverage diverse methodological approaches, including large-scale longitudinal studies, quasi-experimental designs, and randomized controlled trials, to elucidate the complex biological mechanisms linking environmental exposures to psychosomatic disorders, as well as to identify modifiable factors. Such evidence will support the prioritization and formulation of public health strategies targeting modifiable environmental and lifestyle risk factors, ultimately aimed at reducing the global burden of psychosomatic disorders.
